# Establishment and Characterization of Cell Lines from Canine Metastatic Osteosarcoma

**DOI:** 10.3390/cells13010025

**Published:** 2023-12-21

**Authors:** Ya-Ting Yang, Alexander I. Engleberg, Vilma Yuzbasiyan-Gurkan

**Affiliations:** 1 Department of Small Animal Clinical Sciences, College of Veterinary Medicine, Michigan State University, East Lansing, MI 48824, USA; yangyat1@msu.edu (Y.-T.Y.); engleber@msu.edu (A.I.E.); 2 Department of Microbiology & Molecular Genetics, College of Veterinary Medicine, Michigan State University, East Lansing, MI 48824, USA

**Keywords:** osteosarcoma, metastasis, cytokines, cell lines, transcriptome, canine IO panel

## Abstract

Despite the advancements in treatments for other cancers, the outcomes for osteosarcoma (OSA) patients have not improved in the past forty years, especially in metastatic patients. Moreover, the major cause of death in OSA patients is due to metastatic lesions. In the current study, we report on the establishment of three cell lines derived from metastatic canine OSA patients and their transcriptome as compared to normal canine osteoblasts. All the OSA cell lines displayed significant upregulation of genes in the epithelial mesenchymal transition (EMT) pathway, and upregulation of key cytokines such as CXCL8, CXCL10 and IL6. The two most upregulated genes are MX1 and ISG15. Interestingly, ISG15 has recently been identified as a potential therapeutic target for OSA. In addition, there is notable downregulation of cell cycle control genes, including CDKN2A, CDKN2B and THBS1. At the protein level, p16^INK4A^, coded by CDKN2A, was undetectable in all the canine OSA cell lines, while expression of the tumor suppressor PTEN was variable, with one cell line showing complete absence and others showing low levels of expression. In addition, the cells express a variety of actionable genes, including KIT, ERBB2, VEGF and immune checkpoint genes. These findings, similar to those reported in human OSA, point to some genes that can be used for prognosis, targeted therapies and novel drug development for both canine and human OSA patients.

## 1. Introduction

Osteosarcoma (OSA), the most common bone tumor, is a highly aggressive tumor that occurs naturally in both humans and dogs. In humans, OSA (hOSA) is a rare tumor compared to other tumor types, and the incidence rate is around 1/100,000 [1]. Canine OSA (cOSA), however, has a 14 times higher incidence rate compared to hOSA, around 14/100,000 [2,3]. In canine OSA, several large breeds have higher incidence rates, including the Irish wolfhound, Leonberger, greyhound and German shepherd, with the lifetime risk as high as 8% in the Irish wolfhound and 3% in the Leonberger, from a published population-based study containing 11,350 dogs [4]. Canine osteosarcoma is a unique translational model for human OSA for several reasons. First, cOSA is a naturally occurring disease and is similar in clinical presentation and histopathology to the human disease, with the long bones being common sites of presentation in both species. Second, cOSA presents with a higher incidence rate and high metastatic potential, which makes cOSA a useful tool to investigate treatments for the most challenging disease state. Importantly, heterogeneity, a common and challenging feature of most cancers, is also observed in OSA in both species. The current strategies to treat metastatic OSA are still not effective. Previously, we reported using patient-derived cell lines to identify potential therapeutic combinations in cOSA [5]. Therefore, cell lines generated from patients, especially from a metastatic site, are useful tools in experimental and drug studies [6].

The major cause of death in OSA patients is due to metastatic lesions, and the most common site is lung metastases. Metastasis in osteosarcoma patients leads to shorter overall survival and progression-free survival compared to no-metastasis patients in both humans [7] and canines [8]. For the past forty years, despite advances in treatments for other cancers, the overall survival rate for OSA has stagnated. This is in part due to the low number of OSA cases in humans, the high cost of drug development and the difficulty in carrying out clinical trials with novel agents on a small patient population dispersed across large distances. In order to gain a better understanding of OSA biology, and to facilitate drug development, studies have used commercially available human OSA cell lines and patient-derived OSA cell lines from dogs [9,10,11,12] and humans [6,13,14,15,16] and evaluated OSA xenograft models [13,15]. The major challenge in OSA, as in many other cancers, is the treatment of metastatic disease. In the current study, we established three novel canine OSA cell lines, BR (a mongrel), BZ (German shepherd) and LK (Doberman pinscher), from metastatic sites in patients that were previously treated with chemotherapy before tumor recurrence. We selected osteoblast cells as our controls. As there is only one commercially available canine osteoblast source, CnOB, we isolated osteoblasts from bone tissue from two normal dogs and established osteoblast cell strains in our laboratory. We then investigated the expression of cancer related genes using the NanoString nCounter^®^ Canine IO (immuno-oncology) panel.

## 2. Materials and Methods

### 2.1. Origins of Tumor and Normal Cells and Cell Culture

All three canine OSA cell lines were established from discarded tissue from patients presented to the Michigan State University Teaching Hospital (Table 1), with IACUS approval. (1) The BR cell line was established from a metastatic site in the lungs. (2) The BZ cell line was established from a fine-needle aspirate of a metastatic mass under the right eye on the zygomatic arch from an eight-year-old male German shepherd dog with osteosarcoma. The patient had received two cycles of carboplatin after diagnosis of primary osteosarcoma lesion on the right femur. (3) The LK cell line was established from a metastatic lesion in the lung of a three-year-old male Doberman Pinscher. The patient had received two cycles of carboplatin (and two cycles of doxorubicin, one cycle of Cytoxan) after the primary osteosarcoma was found on left distal radius. The diagnosis of OSA was based on the histopathology findings. The cell line was established, pelleted, fixed in formalin and embedded in paraffin and examined by histopathology. Positive staining for osteocalcin (OC) and vimentin also confirmed OSA. Canine osteoblast strain (CnOB) was purchased from Cell Application Inc (San Diego, CA, USA). Aside from CnOB cell strain, two other canine osteoblast cell strains, MSUCnOB1 and MSUCnOB2, were derived from normal dog bones from two 13-week-old beagles. These three osteoblast cell strains were used as controls in the NanoString studies. All canine osteoblast strains were verified via quantitative real-time PCR (RT-PCR) of expression of common osteoblast markers, osteocalcin and collagen type I alpha chain (COL1A1), as described in our previous work [17], and results are presented in Supplemental Figure S1. Osteoblast cell strains were used as controls for the malignant OSA cell lines as osteoblasts have been demonstrated to be the cells of origin for malignant transformation in OSA, as reviewed [18].

### 2.2. Preparation and Maintenance of Cell Culture

The BZ cells were isolated from a fresh fine-needle aspirate, first treated with RBC lysis buffer for 10 min and pelleted. Cells were then resuspended in Minimum Essential Medium α (αMEM medium, Gibco, Carlsbad, CA, USA) supplemented with 10% fetal bovine serum and antibiotics (0.1% gentamycin, Life Technologies, Carlsbad, CA, USA) and seeded to a 100 mm cell culture dish. The BR and LK cells were isolated from tumor masses. The tumors were minced into small pieces and digested with type 2 collagenase (1 mg/mL, Gibco, Carlsbad, CA, USA) in a 37 °C incubator for 3 h. After the digestion, cells were filtered through a strainer and put into 2D cell culture with complete medium (90% αMEM medium supplemented with 10% fetal bovine serum, and gentamicin). The OSA cell line was passaged using 0.05% trypsin when cells reached 80% confluence. NIH3T3 (obtained from ATCC, Manassas, VA, USA) is a mouse fibroblast cell line that we included as a positive control for PTEN expression study in Western blotting. The human OSA cell line SAOS2 (obtained from ATCC, Manassas, VA, USA) was used as a positive control in IHC studies as well a positive control for the protein p16^INK4A^ in Western blots. Both cell lines NIH3T3 and SAOS2 were maintained in Dulbecco’s Modified Eagle Medium (DMEM medium, Gibco, Carlsbad, CA, USA) supplemented with 10% fetal bovine serum, and 0.1% gentamicin. Canine osteosarcoma cell lines were maintained in αMEM medium supplemented with 10% fetal bovine serum, and gentamicin. Canine osteoblast cells were maintained with CnOb growth medium (Cell Applications, San Diego, CA, USA). All cell lines were incubated at 37 °C in a humidified atmosphere containing 95% air and 5% CO_2_.

### 2.3. Immunohistochemistry Staining of OSA Cells

For immunolabeling, histochemistry staining was performed using the CellSieve™ CTC Enumeration Kit (Creatv MicroTech, Rockville, MD, USA) in accordance with the manufacturer’s protocol. OSA cells were first seeded into a chamber slide (Lab-Tek™) and then fixed with CellSieve™ Prefixation Buffer for 15 min at room temperature and washed with PBS. Next, fixed cells were incubated with CellSieve™ Postfixation Buffer for 20 min and changed to CellSieve™ Permeabilization buffer for 20 min. Cells were washed with PBS after each incubation. Later, two fluorescent dye-conjugated antibodies (osteocalcin/FITC and vimentin/EF615) were added to the chamber and incubated with cells for 2 h. After washing with PBS, a drop of CellSieve™ Mounting Solution with DAPI (4′,6-diamidino-2-phenylindole), was applied on the top of fixed cells. Images of all labeled cells were captured using laser-scanning confocal microscopy with Nikon C2 microscope (Nikon, Melville, NY, USA). For visualization of DAPI, osteocalcin and vimentin, filters were used with excitation/emission wavelengths of 405/445 nm, 488/525 nm and 561/600 nm, respectively. Osteocalcin Alexa Fluor^®^ 488-conjugated antibody was obtained from R&D Systems (Minneapolis, MN, USA (cat#: IC1419G)), and vimentin/eF615^®^ antibody was provided by Creatv MicroTech (Rockville, MD, USA).

### 2.4. OSA Cells and Osteoblasts Doubling Time

To determine the cell population doubling time, cells were plated at a density of 5000–8000 cells/well in a 12-well plate suspended in 1 mL of complete medium in triplicate. Wells from each cell line or cell strain were plated, trypsinized and counted by cell counter (Countess™, Invitrogen, Waltham, MA, USA). The population doubling time was calculated using the following formula:

$$Doubling\;time=\frac{duration*log(2)}{log(FinalConcentration)-log(InitalConcentration)}$$

### 2.5. Migration Assay

The wound healing assay was used to examine the migration capacity of cells in a monolayer. Briefly, 100,000 cells/well were plated overnight and allowed to reach 70–80% confluence in 6-well plates. Before placing scrapes, cells were incubated with serum-free medium overnight and then treated with mitocycin C (5 µg/mL, Sigma-Aldrich, St. Louis, MO, USA) for one hour. Then, a 1 mL pipette tip was used to scrape the monolayer and generate wounds under aseptic conditions. The cells were then incubated with cell culture medium with 1% FBS. Each wound area was photographed after being made, as well as 12 and 24 h thereafter. The scratch area remaining was calculated by analyzing the images at the online website https://sketchandcalc.com/ accessed on 25 May 2023, which uses Gauss’ shoelace formula [19]. Statistical analysis was completed using two-way ANOVA test via Prism 9.0, GraphPad Software.

### 2.6. Gene Expression Profiling of BR, BZ and LK Cells

Total RNA of BR, BZ, LK and osteoblast cells (passage 3 to passage 5) were extracted using the mirVana miRNA Isolation Kit, with phenol (ThermoFisher Scientific, Waltham, MA, USA), then quantified by Qubit™ (ThermoFisher Scientific, Waltham, MA, USA). The RNA quality was evaluated by Agilent 4200 TapeStation (Agilent Technologies, Santa Clara, CA, USA). Gene expression was evaluated in triplicate by using the nCounter^®^ Canine IO panel (NanoString Technologies, Seattle, WA, USA) according to the standard NanoString protocol [20]. The panel includes a total of 800 genes across 47 pathways involved in cancer and immunity, as listed at the NanoString website [21].

### 2.7. Gene Expression and Pathway Analysis Using the ROSALIND^®^ Platform

Raw output from the nCounter^®^ Canine IO panel (NanoString Technologies, Seattle, WA, USA) was uploaded to the ROSALIND^®^ (ROSALIND, San Diego, CA, USA) online software platform (https://www.rosalind.bio (accessed on 11 May 2023)) for further gene expression and pathway analysis [22]. Twenty housekeeping genes were selected using the geNorm algorithm with the NormqPCR R library for calculation of normalization, fold changes and *p*-values. NanoString gene expression values were normalized using the 20 reference genes included in the panel [23]. The “fast method” as described in the nCounter Advanced Analysis 2.0 User Manual was used to calculate fold changes and *p*-values. The Benjamini–Hochberg method of estimating false discovery rates (FDR) was used for *p*-value adjustment. Heatmap gene clustering was performed using the PAM (Partitioning Around Medoids) method via the fpc R library [24]. Hyper-geometric distribution was employed to analyze pathway enrichment, domain structure and gene and other ontologies. Similarities and dependencies between Gene Ontology (GO) terms from the topGO R library were used to perform Elim pruning correction [25]. A variety of database sources were referenced for use in enrichment analysis, including Interpro [26], NCBI [27], MSigDB [28,29], REACTOME [30], WikiPathways [31].

### 2.8. Validation of NanoString Analysis

Relative quantitation of gene expression was additionally carried out by reverse transcription and amplification in a QuantStudio™ 3 Real-time PCR system (ThermoFisher Scientific, Waltham, MA, USA) for 10 genes (5 with high and 5 with low expression) and analyzed with relative quantification using GAPDH as an internal control. A total of 2000 ng of each RNA sample was treated with TURBO DNA-*free*™ kit (Invitrogen, Waltham, MA, USA), inactivated and reverse transcribed using random hexamer primers (Promega, Madison, WI, USA) and reverse transcriptase superscript III (ThermoFisher Scientific, Waltham, MA, USA). For input into each PCR reaction, 50 ng of cDNA was used. Primers and conditions are presented in Supplementary Table S1.

### 2.9. Western Blot Analysis

Western blot analysis was carried out as described before [5]. Briefly, the OSA cells and fibroblasts were lysed with CelLytic M lysis buffer (C2978, Sigma-Aldrich, St. Louis, MO, USA) in the presence of protease inhibitor (P8340, Sigma-Aldrich) and phosphatase cocktail inhibitor B (sc-45045, Santa Cruz Biotechnology, Dallas, TX, USA). Proteins were resolved on Bolt Bis-Tris 4–12% polyacrylamide gels (ThermoFisher Scientific, Waltham, MA, USA) and transferred to polyvinylidene difluoride membranes, incubated with 5% bovine serum albumin (BSA) for 2 h at room temperature, before being incubated with the primary antibodies at 4 °C overnight. The primary antibodies used were PTEN (Cell Signaling Technology, D 4.3, 1:2000), p16^INK4A^ (Santa Cruz Biotechnology, F-8, 1:500) and β-actin (Cell Signaling Technology, 8H10D10, 1:2000. The secondary antibodies were obtained from (LI-COR Biosciences, donkey anti-mouse; goat anti-rabbit (LI-COR Biosciences), both used at a 1:15,000 dilution and incubated with the blot for 1 h at room temperature. The membranes were visualized using the Odyssey^®^ M Imaging System (LI-COR Biosciences, Lincoln, NE, USA) and analyzed using Image Studio™ Lite software 5.2.5 (LI-COR Biosciences, Lincoln, NE, USA).

## 3. Results

### 3.1. Characteristics of Established OSA Cell Lines

Three OSA cell lines from metastatic lesions and two osteoblast cells strains derived from bone tissue of normal dogs were established (Table 1). Tissues that would otherwise be discarded were used for both the normal strains and OSA cell lines with IACUC approval.

**Figure 1:**
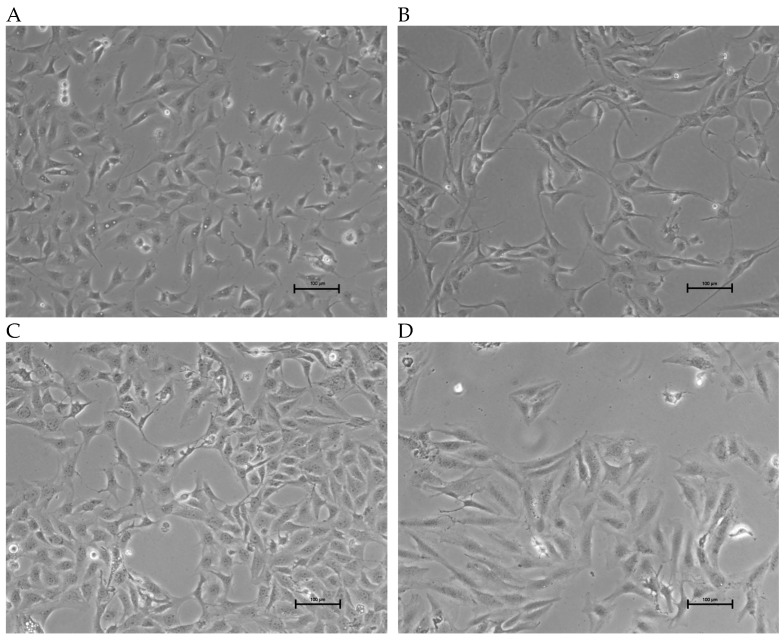
Images of OSA cell lines during culture. Phase contrast images showing growth morphology at 10× magnification. (**A**) BR cells (passage 47); (**B**) BZ cells (passage 45); (**C**) LK cells (passage 35); (**D**) SAOS2; calibration bar —100 μm.

**Figure 2:**
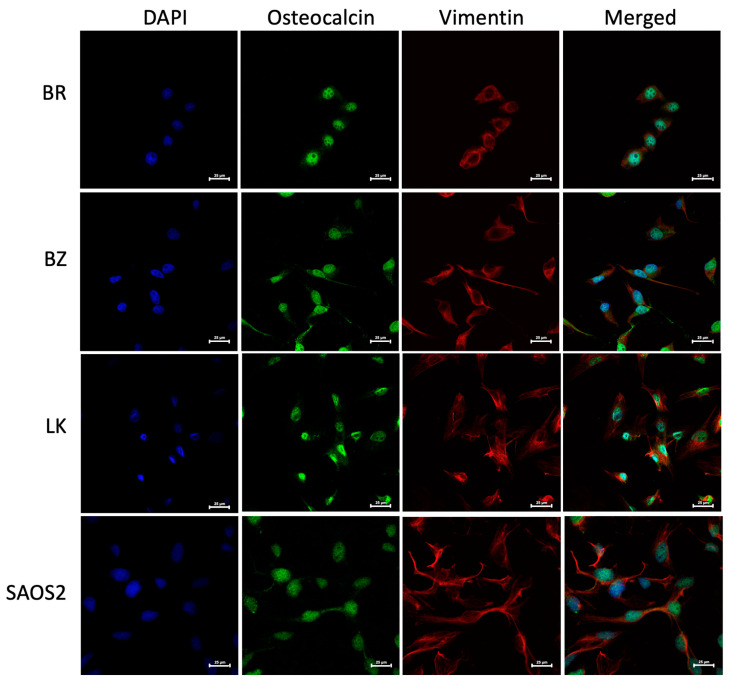
The canine BR, BZ and LK cell lines were confirmed as osteosarcoma by IHC staining. DAPI signal reflects the staining of nuclei in blue, Alexa Fluor^®^ 488 of osteocalcin in green and eF615^®^ of vimentin in red. Images were acquired at 60× magnification with calibration bar —25 μm. The human OSA cell line SAOS2 was used as a positive control. Osteocalcin is a secreted protein and is being produced continuously, sometimes appearing as cytoplasmic, sometimes nuclear and sometimes covering the cell, especially when the cells are small.

**Figure 3:**
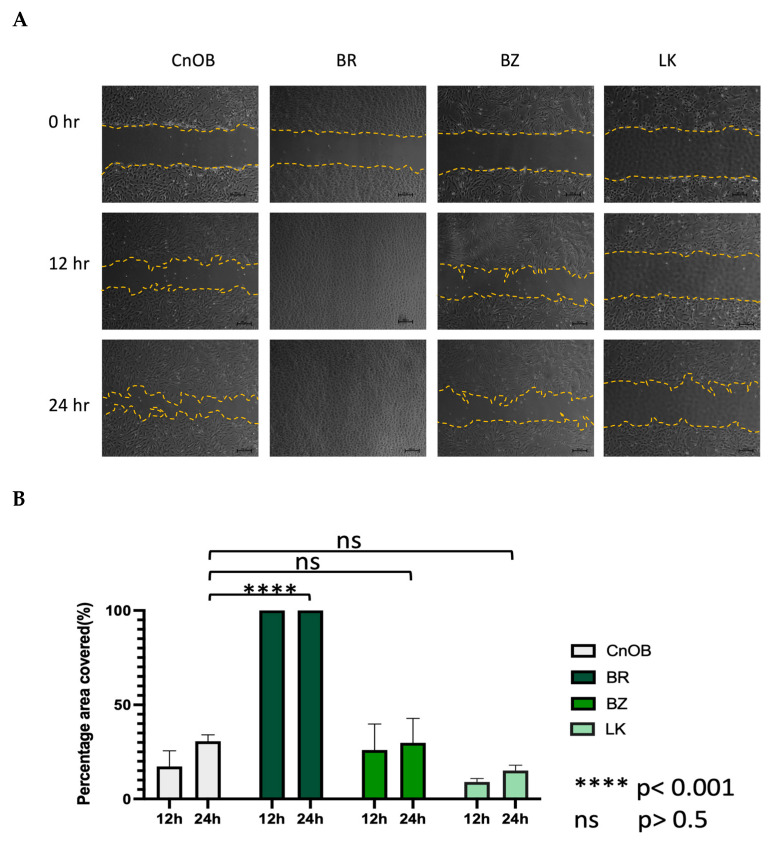
The wound healing assay shows the migration ability variation among OSA cell lines. (**A**) The represented pictures of all cells show that BR cells migrated and filled the wound area within 12 h, while other wounds remained. (**B**) Graphical representation of wound healing assay. Each bar represents the average from three measurements.

The morphology of the three OSA cell lines is spindle-like, as shown in Figure 1, and displays a disorganized cell growth pattern. As shown in Figure 2, the immunohistochemical analysis confirmed that all three of the established cell lines maintained the osteoblast-specific marker osteocalcin and mesenchymal marker vimentin. These two markers continued to be expressed at the 30th passage of the BZ cell line. All three cell lines grew over 50 passages and continued proliferating. The cell doubling times for these cell lines are summarized in Table 2. The doubling times for BR, BZ and LK are 14.8, 20.1 and 16.4 h, respectively. The migration ability of these cells was evaluated by the wound healing assay. As shown in Figure 3, the BR cells migrated and closed the gap faster than the BZ, LK and CnOB cells, which showed similar migration rates.

### 3.2. Normal Canine Osteoblast Cell Strains

Two osteoblast cell strains were established from pieces of rib bone from 13-week-old beagles at euthanasia, carried out for reasons unrelated to this study, and were cultured in the lab. One commercially available strain of primary canine osteoblasts was also used (Cell Applications, San Diego, CA, USA). Cells from passage 3 to passage 5 were used for RNA extraction in this study.

### 3.3. NanoString Gene Expression and Pathway Analysis

**Figure 4:**
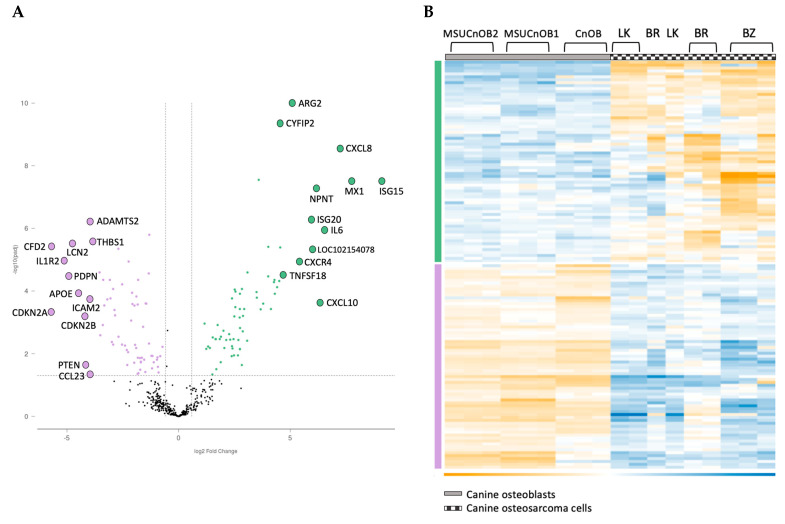
Volcano scatter plot (**A**) and associated heatmap (**B**) of genes in cancer pathways analyzed by nCounter^®^ Canine IO panel. On the volcano plot, dotted vertical lines indicate a 1.5-fold change in normalized gene expression and the dotted horizontal line indicates a p-adjusted value cut-off of 0.05. The overexpressed genes are labeled in green and underexpressed genes are in purple. The 12 most upregulated and the 12 most downregulated genes are labelled for clarity in Figure 4A.

The nCounter^®^ Canine IO panel consists of 800 genes, covering 47 pathways. RCC files from the panel were imported into the ROSALIND^®^ software platform and utilized for analysis of gene expression [20,32]. As shown in Figure 4, unsupervised clustering revealed that the cancer cell lines and the normal osteoblasts displayed distinct gene expression patterns, with cancer cell lines clustering together, as did the normal osteoblasts. Of the 800 genes, 139 were identified to be differentially expressed using a 1.5-fold change threshold in the cancer cell lines as compared to the normal osteoblasts. Among these genes, 69 genes were upregulated (shown in green bar) and 70 genes were downregulated (shown in purple bar) compared to osteoblast cells (Figure 3). All normalized gene expression data can be found in Supplemental Table S2.

Among the 139 genes, the 12 most upregulated and 12 most downregulated genes are listed in Table 3. The most upregulated genes are ISG15 and MX1, both type I interferon response genes, followed by cytokines CXCL8, IL6 and CXCL10. Other significant genes are involved in the epithelial mesenchymal transition (EMT) pathway and the cell cycle. NPNT, the extracellular matrix protein nephronectin, is consistently upregulated in the three canine OSA cell lines. While there is some variability in the degree of the changes, the directionality of change is consistent across all three OSA cell lines.

Ten genes selected from the above table were used in real-time RT-PCR studies as further validation of the NanoString platform. The correlation of gene expression between NanoString and RT-PCR analysis was excellent, with r values ranging from 0.86 to 0.94. The results are presented in Supplementary Table S3.

### 3.4. Epithelial Mesenchymal Transition (EMT) Pathway

**Figure 5:**
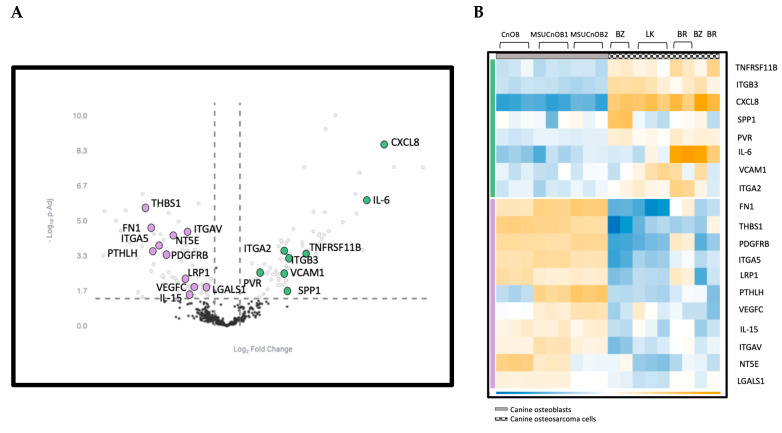
(**A**) Volcano scatter plot of genes in cancer pathways analyzed by nCounter^®^ Canine IO panel. Dotted vertical lines indicate a 1.5-fold change in normalized gene expression and the dotted horizontal line indicates a p-adjusted value cut-off of 0.05. The upregulated genes are labeled in green and downregulated genes are in purple. (**B**) Heatmap of genes in the EMT pathway showing differential expression. Genes in the EMT pathway are highlighted in both panels.

The most significantly affected pathway in the OSA cell line analysis was the EMT pathway, with a p-adjusted value of 0.00048, as indicated in the MSIGDB Pathway Collection [29]. In the EMT pathway, eight genes (TNFRSF11, ITGB3, CXCL8, SPP1, PVR, IL-6, VCAM1 and ITGA2) showed upregulation and eleven (FN1, THBS1, PDGFRB, ITGA5, LRP1, PTHLH, VEGFC, IL-15, ITGAV, NT5E and LGALS1) showed downregulation (Figure 5A). The upregulated genes are indicated with the green bar and downregulated genes with the purple bar (Figure 5B).

### 3.5. Cell Cycle

The three OSA cell lines showed downregulated gene expression of CDKN2A (which encodes p16^INK4a^), CDKN2B, THBS1, PTHLH, CDKN1A, CCND1 and CCND3 and upregulated expression of CXCR4, BIRC5, RAD51, CDKN2C, BCL2, BRCA1, PCNA and CHEK2. CXCR4 is the most upregulated gene, with a fold change of 5.4. The fold changes for CDKN2A and CDKN2B are −5.7 and −4.2, respectively.

### 3.6. Cytokines

The three OSA cell lines showed downregulated gene expression of IL1R2, IL2, JAK2 and IL13RA1 and showed upregulated expression of genes including CXCL8, IL6, CXCL10, CCL5, SPP1, OAS3, HBEGF, CD70 and IL15. CXCL8 and IL6 are the most upregulated genes, with fold changes of 7.2 and 6.5, respectively.

### 3.7. Immune Checkpoints and Targetable Targets on OSA Cells

The complexity and heterogeneity in canine and human osteosarcoma has contributed to the difficulty to treat osteosarcoma patients, indicating the need for individual treatment strategies. For more advanced osteosarcoma patients, the novel treatment options include immune checkpoint inhibitors (PD-1, PD-L1, CTLA4, TIM3 and IDO1) and new druggable targets, such as KIT, ERBB2, VEGF and PDCD1 and others as summarized in Table 4 below.

The gene expression assay pointed out potential therapeutic targets on these OSA cells All three cell lines expressed KIT, ERBB2, VEGF and PDCD1, which are potential targets for novel treatments. Toceranib (Palladia), the first FDA-approved first tyrosine kinase inhibitor for treating dogs with mast cell tumor, targets KIT, VEGFR, FLT3 and PDGFR. ERBB2 is another druggable target among these cells, indicating the potential use of anti-HER2 treatments on canine OSA.

### 3.8. PTEN and p16^INK4A^

**Figure 6:**
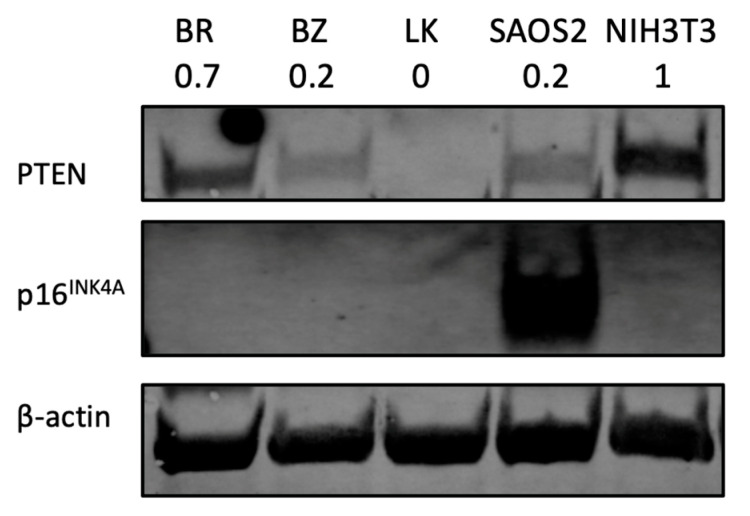
PTEN and p16^INK4A^ protein analysis by Western blot. NIH3T3 is a mouse fibroblast cell line used as a positive control for PTEN expression study. Human OSA cell line SAOS2 is used as a positive control for p16^INK4A^. The number under each cell line designation indicates the PTEN protein band intensity normalized to β-actin and compared to NIH3T3.

As PTEN and CDKN2A (which codes for p16^INK4A^) are among the most frequently downregulated or deleted loci in osteosarcomas, the protein expression of both was assessed by Western blot. The human OSA cell line SAOS2 was used as a positive control for p16^INK4A^ and the mouse fibroblast cell line NIH3T3 was used as a positive control for PTEN protein expression. Beta-actin was used to reflect protein load per lane. As shown in Figure 6, two canine OSA cell lines, BZ and LK, showed PTEN loss, whereas BR had lower PTEN expression.

## 4. Discussion

Osteosarcoma is a highly complex and heterogeneous bone tumor in both humans and dogs. Improvements in OSA treatment have stalled for almost forty years; therefore, novel treatment strategies are needed. One of the critical components for drug discovery studies is the availability of cell lines. Compared to human OSA cell lines, canine OSA cell lines are very limited and often difficult to obtain. In the current study, we derived and characterized three canine OSA patient cell lines from metastatic sites and characterized them, comparing gene expression with that of normal osteoblasts derived from three canine sources. NanoSting profiling using the nCounter^®^ Canine IO (immuno-oncology of cancer) panel allowed for study of over 800 genes, with 139 showing differential expression. Not surprisingly, the EMT pathway is the most significantly altered pathway.

Due to the current limitations in treatment options for metastatic OSA patients, discovering potential molecular targets would enhance the development of novel therapeutic strategies. In the current study, we reported the overexpression of cytokines IL-6 and CXCL8 in all three cell lines. Interestingly, in a previous study [60], Cross et al. reported that, in paired samples, IL-6 and CXCL8 were enriched in metastatic tumors compared to primary tumors. Furthermore, the dual inhibition of IL-6 and CXCL8 resulted in significantly less formation of lung metastasis and longer survival time compared to a no-treatment group [60]. Another chemokine receptor, CXCR4, was upregulated in OSA cells in this study, in agreement with previous findings in canine OSA [61,62]. Fan et al. reported that CXCR4 expression was detected in both primary (8/11) and metastatic samples (2/8). Byrum et al. reported that a third-generation amino bisphosphonate, zoledronate, was able to inhibit CXCR4 expression in OSA cells and in canine patients. In the canine patient cohort, zoledronate decreased circulating CXCR4 concentrations in 18 of 20 dogs with OSA [62]. Further work to thoroughly characterize the influence of these cytokines on the behaviors of the cells is needed. Interestingly, in a study of 54 primary canine OSA subjects [63], two clades were identified that differed significantly with regard to expression of genes involved in the cytokine pathway, including changes in expression regarding VCAM1, CDKN1A, SERPING1, IFIT2, ICAM2, TLR3, TNFRSF11A and ISG15 [63]. However, information on the clinical course of these cases was not available, and it would have been interesting to see if these cases had greater metastatic potential, an aspect that requires further study.

The top two upregulated genes among the three OSA cell lines, ISG15 and MX1, are type 1 interferon response genes (Table 3). While the role of ISG15 or MX1 in osteosarcoma is not well-studied, a recent study identified ISG15 as a potential therapeutic target in human OSA patients [64]. ISG15 is highly expressed in metastatic OSA compared to primary sites [65] and promotes the proliferation, migration and invasion of OSA cells [64]. In addition, immune cell infiltration levels are altered by the expression of ISG15 in human OSA cells [64]. MX1 has been identified as an independent prognosis indicator in breast cancer [66] and head and neck cancer [67]. A dichotomous role for interferon response genes has been put forth [67] where stimulation by MX1 and ISG15 may result in T-cell exhaustion rather than stimulation. Taken together, our data support a role of ISG15 and MX1 in canine osteosarcoma.

NPNT, one of the top upregulated genes (Table 3) among OSA cells, was identified. While the role of NPNT in osteosarcoma has not been previously reported on, in human breast cancer, high NPNT is associated with bone [68] and brain metastasis [69] and poor prognosis, especially with regard to metastasis-free survival [68,69]. NPNT has been implicated in increased MAPK signaling, stimulation of osteogenesis and in the EMT [68,70]. Therefore, it is possible that a high level of NPNT may be associated with increased incidence of metastases and poor prognosis in OSA patients as well.

One of the major findings is the downregulation of CDKN2A (encoding p16^INK4A^) in all three OSA cell lines. We also evaluated p16^INK4A^ and showed decreased expression in the Western blot study. The p16^INK4A^ is a tumor suppressor that is frequently lost in human osteosarcoma, but its role as an independent prognostic biomarker remains controversial [71,72]. In previous studies, downregulation of p16^INK4A^ is also detected in canine OSA tissue and showed potential as a prognostic indicator [73,74]. Genome-wide studies identified 33 regions associated with canine osteosarcoma in several highly heritable breeds, including the greyhound, Rottweiler, Irish wolfhound [75] and Leonberger dog [76], and they include CDKN2A as one of the risk loci.

PTEN loss is documented in many cancers, including both human [77] and canine OSA [74,78,79]. One of our cell lines, LK, showed a significant PTEN reduction in the gene expression assay. The result of PTEN absence was confirmed by Western blotting (Figure 6). LK showed PTEN loss, while BR and BZ showed lower PTEN expression compared to normal control NIH3T3 cells. Positive PTEN expression is associated with a higher 5-year survival rate compared to negative PTEN patients [80]. Aside from being a clinical predicator, PTEN is also a potential therapeutic target for in vitro studies. Activators of PTEN such as tepoxalin [81], evodiamine [82] or celecoxib [83] have been tested, confirming the importance of PTEN regarding human OSA [84].

As outcomes of chemotherapies on OSA patients have not improved during the past 40 years, other therapeutic strategies are being evaluated in OSA patients, including immune checkpoint inhibition. Checkpoint inhibition, alone or in combination, has been helpful in some studies in other cancers but has only been started to be used in OSA. One example is the use of programmed death-1 (PD1) blockade with pembrolizumab alone in a study of 12 human advanced OSA patients. After a median of two cycles of pembrolizumab treatment, the median progression-free survival was 1.7 months and overall survival was 6.6 months [59]. While this was a disappointing end to the study, it should be noted that PDL1 expression was strong in only one of the samples, and that patient showed the best response [59]. In another study of pembrolizumab in various sarcomas, twenty-two OSA patients were included, and only one patient with metastatic OSA responded to the treatment with more than 50% tumor shrinkage and durable response for over 6 months [85]. It is noteworthy that none of the OSA patients in this study expressed PD1 [85]. Other researchers have investigated the potential of combining two checkpoint inhibitors, ipilimumab, targeting CTLA4, and PD1 blockade, with nivolumab. A total of seventy-six patients with metastatic sarcoma were included in this phase 2 trial, where 16% (six of thirty-eight) of the patients receiving the combination treatment showed a positive response (four showed more than a 30% decrease in tumor size and two showed a complete response, with total disappearance of the tumor) [86]. In contrast, only 5% (two of thirty-eight patients) in the PD1 inhibitor group showed a positive response of more than a 30% decrease in tumor size [86]. The expression of immune checkpoint genes in the tumor or the tumor micro-environment was not reported in the above study [86]. Given the various responses of immune checkpoint inhibitors among OSA patients, it will be important to understand the characteristics of the patients and their tumors to identify the patients that can benefit from the treatments. However, the expression of checkpoint proteins in OSA tissues in the cells in the tumor micro-environment is not well-studied. Intertumoral heterogeneity must be appreciated in the design of clinical studies and should guide rational approaches to treatments.

Targeting HER2 in OSA patients is another approach. A previous study of monoclonal antibody Trastuzumab as an adjuvant therapy showed no clinical benefit in HER2-overexpressed OSA patients; the overall survival rate for 30 months is 59% in HER2-overexpressed patients and 50% in a patient group with overexpression [56]. However, a recombinant *Listeria* vaccine expressing a chimeric human HER2/neu construct in a canine osteosarcoma trial showed prolonged overall survival time, with 1-, 2- and 3-year survival rates of 77.8%, 67% and 56% in 18 treated canine patients compared to 55%, 28% and 22% in the historical controls, respectively [87]. In addition, an EGFR/HER2-targeting vaccine, utilizing a portion of the canine EGFR (p527-545), a conserved extracellular domain of EGFR and homologous to HER2 and HER3 extracellular domains, along with vaccination for borrelia burgdorferi antigen as an adjuvant, has been tested in 43 dogs with appendicular OSA overexpressing EGFR and showed some positive results. In that study, 65% of the patients that received EGFR/HER2 immunization after standard of care survived over 12 months, while the 12-month survival rate with standard of care only was reached by 35% to 40% of the dogs based on the historical literature [57]. While all three of our cell lines express HER2, as well as EGFR, this has not been uniformly studied in dogs or humans. Further characterization of HER2 and EGFR expression in OSA patients is needed for evaluating the benefits of such immunization. Furthermore, combination treatments with checkpoint blockade and other approaches such as tyrosine kinase inhibitors (TKIs) and immune stimulators such as activation of the stimulator of interferon genes (STING) pathway may need to be considered.

Other aspects of heterogeneity were noted in our OSA cell lines. The three canine OSA cell lines showed differences in doubling time (Table 2) and migration ability (Figure 3). Moreover, the three canine cell lines also showed variation in gene expression levels. For example, the BZ cell line showed the highest expression level on the ISG15 gene in terms of a 10.6 log2 fold increase as the other cell lines showed a 3.3 and 4.6 log2 fold change. On the IL6 gene, the three cell lines BR, BZ and LK had log2 fold changes of 5.1, 8.2 and 6.8, respectively. For PTEN expression, two canine OSA cell lines, BZ and LK, showed PTEN loss, whereas BR had lower PTEN expression. These differences in gene expression reflect further heterogeneity within canine OSA tumors.

These cell lines also increased the diversity of OSA in cell lines in the research field. The most frequently studied OSA commercial cell line is D17 (CCL-183), which was derived from a standard poodle [88]. However, large breeds like the Irish wolfhound, Leonberger, greyhound, German shepherd, Rottweiler and Doberman have a much higher incidence rate of OSA compared to other breeds [89]. Another resource for canine OSA cell lines, Kerafast (Kerafast, Boston, MA, USA), only accounts for six OSA cell lines. The BZ cell line was derived from a German shepherd, and LK was derived from a Doberman, both breeds having a high incidence of OSA [89]. In humans, widely studied cell lines like SAOS2, MG63 and U2OS were all derived from Caucasian patients. Other studies have established OSA cell lines from different ethnic groups, such as Chinese [16] and Japanese [90] patients. The individual and ethnic differences in humans and breed differences in dogs may point out different genetic backgrounds and result in different clinical outcomes. Thus, it is necessary to develop more cell lines for research use, as well as to expand the knowledge of OSA between different ethnic groups and breeds. Future in vivo xenograft studies of these cell lines as well as studies of their in vitro behavior under different conditions, such as under hypoxic conditions, are needed.

Some limitations of our study come from the limited number of genes in the targeted gene panel, which might not include all the relevant genes or pathways related to osteosarcoma. Another limitation is the lack of study of microRNAs, which can also be critical regulators in osteosarcoma, as in other cancers. For example, previous findings from Fenger et. al. pointed out that MiR-9 is overexpressed in canine OSA tumor samples and cell lines [91]. However, the ease of use and high sensitivity and reproducibility of the nCounter^®^ Canine IO panel enable assessment of many genes and key pathways, and it can be a useful tool to integrate into clinical studies [20].

In summary, we described here the establishment of three canine metastatic OSA cell lines, BR, BZ and LK, from a mixed breed dog, German shepherd and Doberman, respectively, and profiled them with the Canine IO panel. To our knowledge, there are no other OSA cell lines established from the Doberman breed. Our findings support further study of immunomodulatory signals in OS patients for both prognosis and drug development and validate the utility of the canine metastatic cell lines and canine patients for further understanding and translational studies for human OSA.

## 5. Conclusions

Our results support the importance of genes in the EMT pathway in metastatic osteosarcoma and further support the relevance of canine osteosarcoma for translational studies in human OSA, such as drug development and testing novel therapeutic agents. Our data demonstrate molecular insights into the three newly developed canine OSA metastatic cell lines. Further exploration of these genes in the clinical setting can lead to the development of prognostic biomarkers as well as novel drug treatments for canine and human osteosarcoma.

## Figures and Tables

**Table 1:** Canine osteosarcoma cell lines.

Cell Line	Sex	Age	Breed	Location	Note
BR	N/A	N/A	Mongrel	Lung metastasis	N/A
BZ	Male	8 years	German shepherd	Zygomatic arch	Underwent two cycles of carboplatin
LK	Male	3 years	Doberman pinscher	Lung metastasis	Euthanized 4 months after diagnosis with OSA. This patient underwent two cycles of carboplatin, one cycle of cyclophosphamide and two cycles of doxorubicin.

**Table 2:** Cell doubling time for three cell lines and osteoblasts.

Cell Line	Doubling Time (Hours)
BR	14.8
BZ	20.1
LK	16.4

**Table 3:** Top 12 upregulated and downregulated genes in canine osteosarcoma cell lines. All shown in log2 fold change as compared to the control osteoblast cell strains.

Name	Log2 Fold Change	p-Adj	BR	BZ	LK
ISG15	9.1	3.00 × 10^−8^	3.3	10.6	4.6
MX1	7.7	2.87 × 10^−8^	3.1	9.2	3.4
CXCL8	7.2	2.54 × 10^−9^	7.2	7.6	6.6
IL6	6.6	1.11 × 10^−6^	7.6	4.1	2.5
CXCL10	6.3	0.000259	5.1	8.2	6.8
NPNT	6.1	5.31 × 10^−8^	5.9	5.1	6.9
LOC102154078	6	4.70 × 10^−6^	7.4	4.2	3.1
ISG20	5.9	5.48 × 10^−7^	4.9	6.4	4.8
CXCR4	5.4	1.22 × 10^−5^	6.5	4.6	3
ARG2	5	5.62 × 10^−12^	4.5	4.7	5.6
TNFSF18	4.7	3.22 × 10^−5^	5	5.3	2.5
CYFIP2	4.6	4.48 × 10^−10^	4.2	5.1	4.2
THBS1	−3.8	2.48 × 10^−6^	−2.8	−5.6	−4.3
ICAM2	−3.9	0.000172	−3.6	−4.6	2.2
CCL23	−3.9	0.04431	−2.9	−4.4	−4.4
ADAMTS2	−3.9	5.48 × 10^−7^	−3	−5.5	−4.1
PTEN	−4.1	0.026195	−0.2	−2.4	−9.8
CDKN2B	−4.2	0.000564	−6	−4.3	−6
APOE	−4.5	0.000116	−2.7	−5.7	−4.7
LCN2	−4.7	3.28 × 10^−6^	−4.8	−6	−3.9
PDPN	−4.9	3.10 × 10^−5^	−7.6	−3.6	−8.1
IL1R2	−5.1	1.05 × 10^−5^	−4.7	−5.2	−5.8
CFD	−5.7	3.71 × 10^−5^	−5.2	−6	−6
CDKN2A	−5.7	0.000478	−6.5	−3.8	−7

**Table 4:** Expression of immune checkpoint genes and druggable targets by OSA cell lines. All shown in log2 fold change.

Name	Alias	OSA avg	BR	BZ	LK	Current Clinical Trial/Reference in Literature
CD27	-	1.7	2	1.7	1.3	Ansell [33]
CD28	-	0.8	1	1.2	0.2	Tang [34]
CD40LG	CD154|TNFSF5	0.9	1.1	1.6	0.1	Tan [35]
CD40	TNFRSF5	5.3	5.3	5.5	5	Samant [36]
IL2RB	CD122	1.7	1.8	2.2	1.3	Judge [37]
TNFRSF9	CD137, 4-1BB	1.3	1.2	2.1	0.7	Segal [38]
TNFRSF4	OX40	3.1	3.1	3.1	3.1	Ruiz [39]
TNFRSF18	GITR	3.1	3.1	2.9	3.4	Ammons [40]
ICOS	-	0.7	1.2	1.3	−0.3	Patel [41]
ADORA2A	A2AR	7.3	6.6	8.6	6.8	Lim [42]
CD276	B7-H3	6.8	7.4	5.8	7	Zhang [43]
BTLA	-	1.6	1.8	1.6	1.5	Schilder AACR meeting [44]
CTLA4	CD152	3	3.2	3	2.6	Mason [45]
IDO1	INDO	4.5	2.3	8.3	2.9	Ikeda [46]
IDO2	INDOL1	2.3	2.4	2.4	2.2	Peng [47]
LAG3	-	3.1	3.4	2.9	3	Ligon [48]
CYBB	NOX2	2	2	2.3	1.6	Chen [49]
CD274	PD-L1	3.6	4.1	3.6	3	Felip E [50]
HAVCR2	TIM3	3.3	3.1	3.3	3.5	Cheng [51]
VSIR	VISTA	5.5	5.8	5.2	5.5	Zong [52]
KIT	c-KIT	3.1	4.2	2.9	2.3	Kim [53]
VEGFA	VEGF	9	9.7	7.6	9.6	Sayles [54]
ERBB2	HER-2|c-erbB-2	6.7	7.1	6.6	6.6	Musser [55] Ebb [56]
EGFR	-	7.2	7.7	7.6	6.4	Doyle [57]
PDCD1	PD1	2.8	2.5	3.5	2.4	Igase [58] Boye [59]

## Data Availability

The manuscript and the supplementary files contain all the data.

## Supplementary Materials

The following supporting information can be downloaded at https://www.mdpi.com/article/10.3390/cells13010025/s1, Figure S1: Relative expression of osteocalcin and COL1A1 in derived and commercial canine osteoblast cell strains, and primer sequences. Table S1: List of TaqMan primers used in RT-QPCR validation; Table S2: Normalized gene expression and a zipped archive with raw data count files; Table S3: Comparison of log_2_fold change in gene expression as determined by NanoString vs. RT-QPCR analysis.
