# Supplementary material for: Establishment and Characterization of Cell Lines from Canine Metastatic Osteosarcoma
Source: Cells. 2023 Dec 21;13(1):25. doi: 10.3390/cells13010025 (PMC10778184; doi:10.3390/cells13010025)
Supplement: Supplementary file 1 [file cells-13-00025-s001.zip › Supplementary Figure S1 - Relative expression of osteocalcin and COL1A1.pdf]

A.

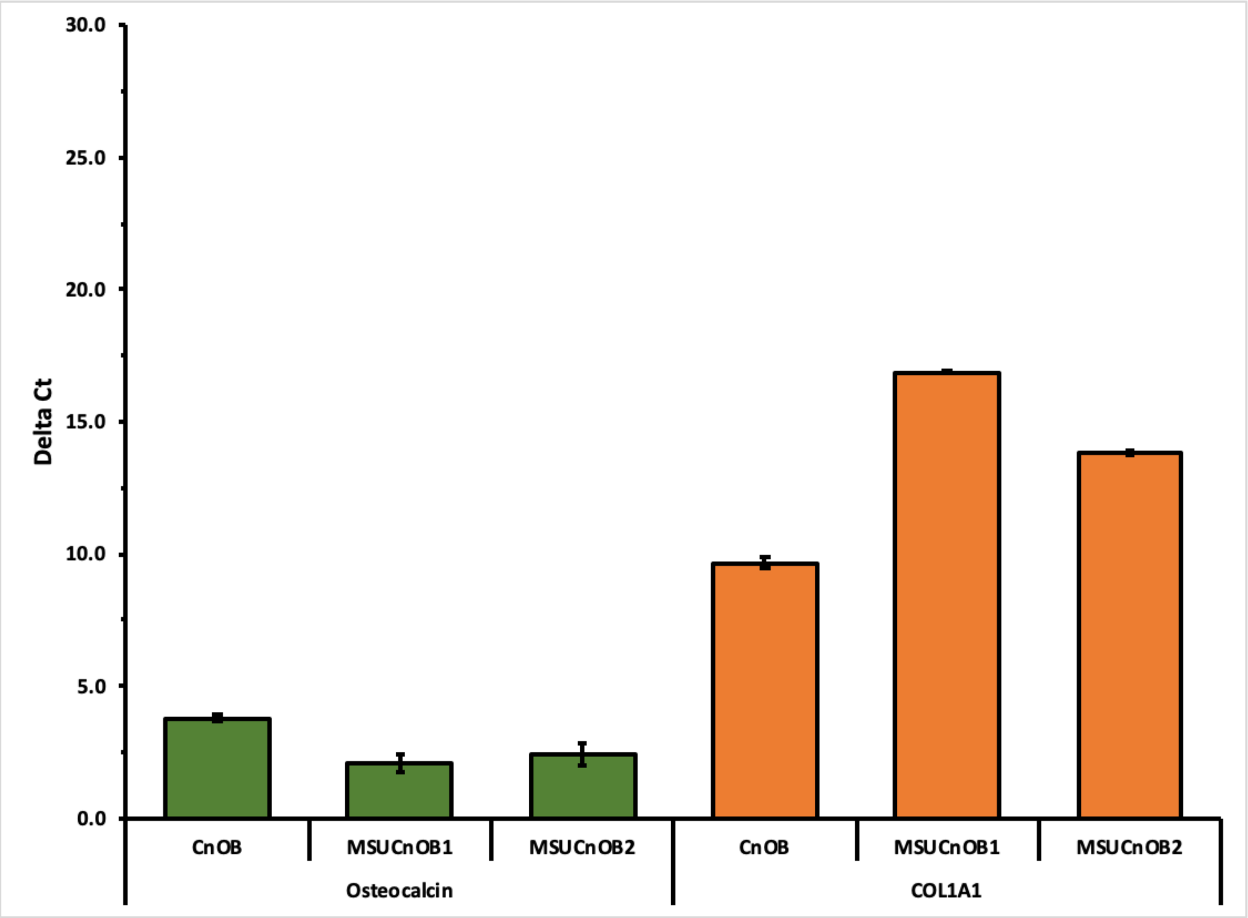

B.

	Forward (5′ -> 3′)	Reverse (5′ -> 3′)
**COL1A1**	GTAGACACCACCCTCAAGAGC	TTCCAGTCGGAGTGGCACATC
**Osteocalcin**	GAGGGCAGCGAGGTGGTGAG	TCAGCCAGCTCGTCACAGTTGG
**Beta Actin (ACTB)**	CTCTTCCAGCCTTCCTTCC	CAGGAGGAGCAATGATCATGA

Supplemental Figure S1. Relative expression of osteocalcin and COL1A1 in derived and commercial canine osteoblast cell strains (**A**); and primer sequences for COL1A1 and osteocalcin (**B**). Osteocalcin and COL1A1 were used for validation of derived canine osteoblast cell strains via real-time PCR (RT-PCR). Gene expression values for osteocalcin and COL1A1 were normalized to expression of ACTB (beta-actin). Osteocalcin and COL1A1 were expressed in all osteoblast cell strain samples.

Quantitative RT-PCR was performed using SYBR™ Green (ThermoFisher Scientific, Waltham, MA, USA) to detect amplification of osteocalcin, COL1A1, and ACTB. Annealing temperature for all primers was 62˚C using the QuantStudio™ 3 Real-time PCR system (ThermoFisher Scientific, Waltham, MA, USA). Samples were tested in triplicate, with error bars depicting standard deviation. All signals were detected at less than 31 cycles.
