# Supplementary material for: Establishment and Characterization of Cell Lines from Canine Metastatic Osteosarcoma
Source: Cells. 2023 Dec 21;13(1):25. doi: 10.3390/cells13010025 (PMC10778184; doi:10.3390/cells13010025)
Supplement: Supplementary file 1 [file cells-13-00025-s001.zip › Supplementary Table S1 - TaqMan primers used for gene expression validation is listed with Assay ID.pdf]

Supplementary Table S1. TaqMan primers used for gene expression validation is listed with Assay ID. Quantitative RT-PCR was performed using TaqMan™ (ThermoFisher Scientific, Waltham, MA, USA) to detect amplification of ISG15, MX1, CXCL8, IL6, CXCL10, CCL23, PTEN, CDKN2B, IL1R2, and GAPDH. Annealing temperature for all primers was 60˚C using the QuantStudio™ 3 Real-time PCR system (ThermoFisher Scientific, Waltham, MA, USA). Samples were tested in triplicate, with error bars depicting standard deviation.

Primer name	Assay ID
ISG15	Cf02644424_m1
MX1	Cf02624057_m1
CXCL8	Cf02624283_m1
IL6	Cf02624153_m1
CXCL10	Cf02622528_m1
CCL23	Cf02644972_m1
PTEN	Cf02690631_m1
CDKN2B	Cf03811587_m1
IL1R2	Cf02647568_m1
CFD	Cf02648771_mH
GAPDH	Cf04419463_gH
